# Antimalarial Agents as Therapeutic Tools Against Toxoplasmosis—A Short Bridge between Two Distant Illnesses

**DOI:** 10.3390/molecules25071574

**Published:** 2020-03-30

**Authors:** Alina Secrieru, Inês C. C. Costa, Paul M. O’Neill, Maria L. S. Cristiano

**Affiliations:** 1 Center of Marine Sciences, CCMAR, Gambelas Campus, University of Algarve, UAlg, 8005-139 Faro, Portugal; asecrieru@ualg.pt (A.S.); a52917@ualg.pt (I.C.C.C.); 2 Department of Chemistry and Pharmacy, Faculty of Sciences and Technology, FCT, Gambelas Campus, University of Algarve, UAlg, 8005-139 Faro, Portugal; 3 Department of Chemistry, University of Liverpool, Liverpool L69 7ZD, UK; pmoneill@liverpool.ac.uk

**Keywords:** *Toxoplasma gondii*, *Plasmodium* spp., antimalarial drugs, drug repurposing, quinolines, endochin-like quinolones, endoperoxides, pyrazolo[1,5-*a*]pyrimidines, antimicrobial peptides

## Abstract

Toxoplasmosis is an infectious disease with paramount impact worldwide, affecting many vulnerable populations and representing a significant matter of concern. Current therapies used against toxoplasmosis are based essentially on old chemotypes, which fail in providing a definitive cure for the disease, placing the most sensitive populations at risk for irreversible damage in vital organs, culminating in death in the most serious cases. Antimalarial drugs have been shown to possess key features for drug repurposing, finding application in the treatment of other parasite-borne illnesses, including toxoplasmosis. Antimalarials provide the most effective therapeutic solutions against toxoplasmosis and make up for the majority of currently available antitoxoplasmic drugs. Additionally, other antiplasmodial drugs have been scrutinized and many promising candidates have emanated in recent developments. Available data demonstrate that it is worthwhile to explore the activity of classical and most recent antimalarial chemotypes, such as quinolines, endoperoxides, pyrazolo[1,5-*a*]pyrimidines, and nature-derived peptide-based parasiticidal agents, in the context of toxoplasmosis chemotherapy, in the quest for encountering more effective and safer tools for toxoplasmosis control or eradication.

## 1. Introduction

Infectious diseases continue to represent leading causes of the global burden at social, economic, and public health levels [1,2,3,4]. Several parasite-borne infectious diseases are especially threatening because of their global impact, including malaria, leishmaniases, and toxoplasmosis. Due to its extended burden across several millennia, malaria has duly benefitted from intense research during the last century and, in view of the spread of parasites’ resistance against antimalarials in use, the efforts must continue [5,6]. Malaria-related research has enabled a better understanding of various aspects attributed to the disease, namely those associated with transmission, infection, and progression inside the human host, including related symptoms and molecular mechanisms, leading to the development of prophylactic measures as well as of structure-based and target-based antimalarial drugs [7]. On the contrary, investments in research related to leishmaniases and toxoplasmosis are far from those accorded to malaria, and most drugs used against these diseases were developed to treat other vector-borne pathologies, including malaria [8]. Toxoplasmosis is a neglected disease, with an urgent need for action since one third of the population is chronically infected with *Toxoplasma gondii* [9].

*Toxoplasma gondii* is a required intracellular protozoan parasite firstly described in 1908 by Nicolle and Manceaux after the observation of an arch-like structure in a rodent, a feature that originated in the designation of the *Toxoplasma* genus (“toxo=arc, plasma=form”) [10]. This parasite affects most warm-blooded mammals; however, its only definitive host known to date is the cat and all other members of the Felidae family. The definitive host is where sexual reproduction takes place, in this case in the cat’s intestine, leading to the formation of infective oocysts. Fecal expulsion of oocysts gives rise to the primary infection route of the parasite’s intermediate hosts, a fecal–oral transmission route through contact with soil, water, or food contaminated with sporulated oocysts [2]. Once inside the intermediate host, the oocysts differentiate into fast-spreading tachyzoite forms of *T. gondii*, which infect all types of nucleated cells, where they reproduce asexually, encysting subsequently in immunoprivileged organs, such as neural and lymphatic tissues, heart, and lungs, under a dormant minimally metabolically active bradyzoite form of *T. gondii* [11,12]. This process consists in a protective mechanism, which allows the parasite to evade the immune system. However, upon failure of the immune system, cysts rupture, releasing bradyzoites into the bloodstream, which rapidly convert into the tachyzoite active form, and reactivating the infection, which usually culminates with a clinical presentation of the disease [12].

Parasite dissemination throughout the intermediate host’s tissues is responsible for the introduction of other transmission routes with high impact to the human host, in which we can include the foodborne transmission of tissue cysts through the ingestion of raw or poorly cooked meats, iatrogenic transmission through organ transplantations and blood transfusions, and vertical transplacental transmission from mother to fetus [13]. 

Healthy individuals infected with this parasite usually do not develop symptoms, because the immune system is able to fight the infection and prevent further outbursts. However, immunodeficient individuals are at high risk of developing severe symptoms, including brain damage, since the brain is the most susceptible organ to primary infections and reactivation of toxoplasmosis. Another concerning matter to take into account is the vertical transmission of the parasite, which, if left untreated, may critically affect the fetus, causing irreversible and possibly fatal consequences [14]. Infection with *T. gondii* acquired during pregnancy or just before conception places the mother at risk of accumulation of tachyzoites in the placenta, which are transmitted to the fetus. Quite interestingly, congenital transmission rates appear to be lower in cases where the mother contracts the parasite in the earlier stages of the pregnancy rather than in the later stages, whereas the consequences to the fetus are far more detrimental in the cases involving an early onset of infection [15]. Specific measures, such as screening tests for early detection of maternal infection and early initiation of treatment, are therefore mandatory for the prevention of vertical transmission.

Furthermore, although infections by encysted bradyzoite forms of *T. gondii* in immunocompetent human hosts have been associated with latency of the disease and an asymptomatic clinical state, recent studies have come to refute such conclusions, connecting these stages of infection with the appearance of alterations in the intermediate host [16,17,18,19,20]. Following alterations in tryptophan metabolism, the human host becomes susceptible to mental disorders, such as schizophrenia, anxiety, and depression [16,17,18]. Studies have also shown behavioral changes in rodents induced by *T. gondii*, namely a decreased fright response to cat odor, as a result of lower renewal levels in host neurotransmitters [20].

Toxoplasmosis is a public health issue that demands much effort towards the implementation of preventive and therapeutic measures in the most vulnerable populations, in order to tackle the infection and prevent the disease from evolving to more severe outcomes. Additionally, novel therapeutic alternatives are necessary to provide more efficient treatment regimens and achieve parasite eradication. The main current shortcomings in the available treatment options are their inefficacy against all of the parasitic stages, namely the acute tachyzoite stage and the chronic bradyzoite stage, where reactivation of the disease becomes a possibility, as happens, for example, in ocular toxoplasmosis and in immunosuppressed individuals, the unequal susceptibility and/or resistance of the parasites to the currently available drugs, and, also, the high prevalence of severe adverse effects from medication to the carrier of the disease [21]. 

## 2. Treatment

### 2.1. Currently Available Drugs

When addressing therapies against infections by *Toxoplasma gondii*, several focal points should be contemplated. Firstly, toxoplasmosis can manifest through different clinical expressions, according to the stage of the infection and the status of the host’s immune system. Given the parasite’s appetence for immunoprivileged tissues, the main clinical presentations occur at ocular or central nervous system (CNS) levels, although pulmonary and multi-organ effects may also develop in severely immunocompromised patients. Additionally, the parasite’s ability to encyst, protecting itself from external agents, makes it difficult to tackle this stage of the disease, especially for cerebral cysts, where the blood–brain barrier emerges as an additional constraint for directed therapies [22].

Treatment guidelines for toxoplasmosis recommend, as first-line therapies for all of the clinical manifestations, a combination between three agents: Pyrimethamine and sulfadiazine, which act synergistically through inhibition of the folate synthetic pathways of the parasite, disrupting its DNA synthesis; and folinic acid, which acts as a coadjuvant in restoring the host’s folate levels [9,22,23]. In hypersensitive patients, protein synthesis inhibitor antimicrobials, such as clindamycin, clarithromycin, azithromycin, or the mitochondrial electron-transport inhibitor atovaquone, may be used as replacements for sulfonamides. The combination of trimethoprim with sulfamethoxazole (co-trimoxazole) is also used because its efficacy was shown to match those of the previously mentioned drugs (Table 1). 

Congenital toxoplasmosis can be approached therapeutically in two distinct ways, whether it is intended for prophylaxis from transplacental transmission or for the treatment of acquired progressive fetal infection, although therapeutic regimens may vary in different geographic regions [15]. Spiramycin is the most recommended drug for a prophylactic approach, usually in the earlier stages of pregnancy where no definitive fetal exams can be performed to confirm the disease. In later stages or in confirmed fetal infection, the first-line combination therapy for toxoplasmosis, consisting of a pyrimethamine/sulfadiazine/folinic acid regimen, is maintained, although a switch to prophylactic spiramycin may also be considered in late-stage infections with negative fetal results [24].

Although currently available drugs allow for elimination of an active infection, these drugs present several side effects, such as renal, hepatic, and hematological toxicity and hypersensitivity [23]. To circumvent these issues and to allow for complete eradication of the parasite, efforts must be made towards the development of novel chemotherapies. Health organizations have adopted a new method of drug development for new purposes based on the investigation of the potential of drugs or drug candidates that are already in use or under development, allowing evasion or minimization of the need for safety tests and drug profile studies. The implementation of such strategies may promote a faster and low-cost expansion of the libraries of promising molecules to combat toxoplasmosis. In this context, the antimalarial arsenal may provide a pool of suitable antitoxoplasmic candidates.

### 2.2. Drug Repurposing in Toxoplasmosis Therapy

#### 2.2.1. Can Antimalarials Treat Toxoplasmosis?

*Toxoplasma gondii* belongs to the family Sarcocystidae and to the phylum Apicomplexa. This phylum includes other pathogens, such as *Plasmodium* spp. (malaria), *Cryptosporidium* spp. (cryptosporidiosis), etc., but the ones that mostly affect human public health are *T. gondii* and *Plasmodium* spp., the causative agents for toxoplasmosis and malaria, respectively. All of the aforementioned pathogens are obligatory intracellular parasites and share a variety of common features. Among them, one which deserves distinctive attention is the apical complex, the hallmark of the Apicomplexa phylum, an indispensable structure for the recognition and subsequent invasion of host cells [25]. Most of these parasites also contain the apicoplast, an essential plastid organelle derived from an endosymbiotic process with a seaweed, which is crucial for the biosynthesis of essential parasitic biomolecules, like fatty acids (type II fatty acid pathway), iron-sulfur clusters, the heme group, and isoprenoid precursors (the non-mevalonate pathway) [25,26]. The high similitude in morphology and biochemistry between *T. gondii* and *Plasmodium* spp. makes them especially interesting from the therapeutic viewpoint, since common metabolic pathways and organelles may serve as targets for the same drugs, as happens with some of the currently available antitoxoplasmic drugs pyrimethamine, sulfonamides, and atovaquone, which were initially designed and developed for the treatment of malaria [27,28]. The apicoplast ribosome inhibitors, such as clindamycin and doxycycline, also fit these criteria, being currently used for the treatment of acute toxoplasmosis and malaria chemoprophylaxis [26]. Hence, drug repurposing stems as a useful approach for drug development in these parasitic illnesses. In fact, several studies focus on the evaluation of antimalarial compounds on *T. gondii* and some even focus concomitantly on therapeutics targeting both parasites [27,29,30,31]. 

Therapies and candidates for toxoplasmosis have been reviewed elsewhere [19,31,32,33,34] and, therefore, for the scope of this work, our aim is to discuss and further explore those classes of compounds that are active and/or promising against infections by *T. gondii*, with a previous or current impact on malaria.

#### 2.2.2. Quinolines

**Figure 1:**
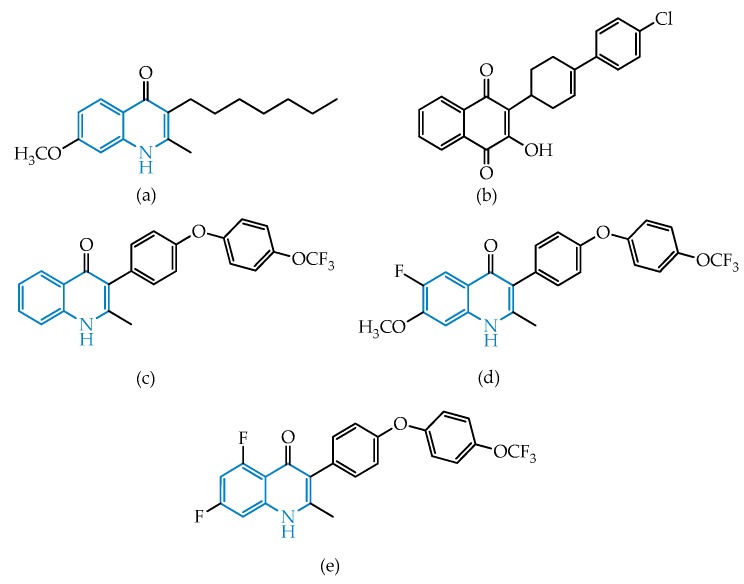
Structural representation of endochin (**a**), atovaquone (**b**), and candidates ELQ-271 (**c**), ELQ-316 (**d**) and ELQ-400 (**e**).

**Figure 2:**
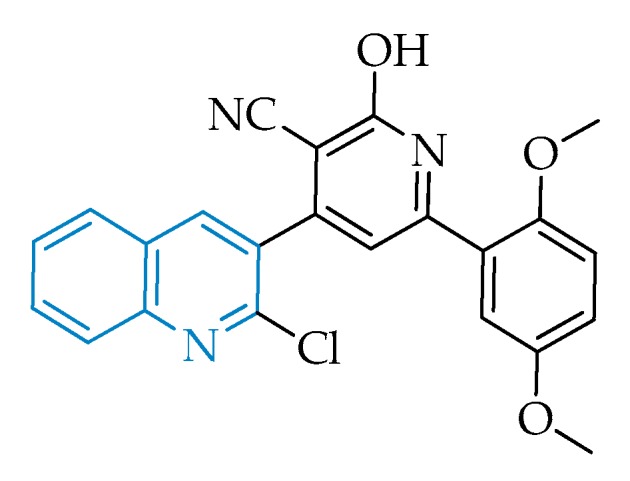
Structural representation of the antitoxoplasmic candidate PPQ-8.

The quinoline scaffold (highlighted in blue in Figure 1 and Figure 2) is a heterocyclic aromatic structure formed by the fusion between a benzene ring and a pyridine ring. Following the isolation of quinine from the bark of the cinchona tree, quinolines were the first class of medicines used against malaria in the western world [35,36,37]. For many years quinoline-based therapeutics were the leading agents in the treatment of malaria, and still remain within the therapeutic options against *Plasmodium* spp. infections nowadays [6]. In addition, the quinoline chemotype offers effective therapeutic solutions for a variety of different diseases, including infections from bacteria, viruses, fungi, parasites, and, in some cases, even in non-infectious illnesses [38]. Quinolines have also been shown to be effective against infections caused by *T. gondii*, in both acute and chronic phases [29,30,32,39,40,41,42]. 

##### Endochin-Like Oxoquinolines (ELQs) and Quinoline-Based PPQ-8

As stated above, quinolines represent one of the major classes of compounds explored for antimalarial chemotherapy. Many quinoline-based drugs were designed and used since the first half of the last century, some of which are still used nowadays against susceptible strains of *Plasmodium*, but the substantial capacity of the parasite to evolve and acquire resistance to those agents required many efforts in developing more potent, less toxic, and well-tolerated molecules [38,43]. Endochin (structure (a), Figure 1) was the first 4-oxo-quinoline (also called quinolone) to be studied in the context of malaria chemotherapy, but, due to its rapid metabolism, it did not fit the criteria to become an effective antimalarial drug [44,45,46,47]. However, the discovery of this compound’s high antiplasmodial activity prompted the use of its core structure as a lead framework in the development of many endochin-like quinolone derivatives [38], targeting parasites’ organelles, namely the mitochondrial ubihydroquinone, A cytochrome *c* oxireductase complex often referred to as the cytochrome *bc1* complex, composed of two catalytically active domains comprising the hydroquinone oxidation (Qo) and the quinone reduction (Qi) sites [48]. This complex is a vital structure for the parasite’s respiratory chain and for the biosynthesis of essential molecules [49], and its relevance in this field grew since it was established as the therapeutic target in *P. falciparum* of currently available antimalarial atovaquone (structure (b), Figure 1) [50]. 

The apicomplexan cytochrome *bc1* complex is highly conserved within the phylum, including in *T. gondii*, with the latter being significantly affected by endochin-like quinolones at both the Qo and Qi sites of this enzyme complex [33,40,41,42]. In fact, ELQs revealed high efficacies, with single-dose cures being observed in some cases, and, as such, some ELQs entered the preclinical phase of development both against toxoplasmosis and malaria [40,51,52]. Promising antitoxoplasmic candidates include ELQ-271 and ELQ-316 (Figure 1; compounds (c) and (d)), which were shown to possibly inhibit the cytochrome *bc1* complex, with high selectivity to the parasitic complex comparatively to the human cytochrome *bc1* complex [41,42]. Regarding the site of action, the authors suggest that ELQ-271 and possibly other endochin-like quinolones inhibit the divergent Qi site, rather than the highly conserved Qo site inhibited by atovaquone [41,42], although structural studies indicate that small changes within the quinolone framework may have a significant impact in the activity of the compounds as well as the binding target [53]. Early docking studies performed in silico at the yeast Qo site of the *bc1* protein complex of *P. falciparum* [54] indicated that both the carbonyl and the N-H group of the 4-oxoquinoline framework are relevant for the potent activity of quinolones. The carbonyl appears to be oriented to enable the formation of H-bonding between the carbonyl and a protonated imidazole nitrogen of His181, while the quinolone N-H is involved in a water-mediated H-bond with the cytochrome b residue Glu272. It is also noteworthy that endochin and the ELQ candidates bear the possibility of quinolone-hydroxyquinoline tautomerism [55], which may impact antiparasitic activity.

Recently, McConnell et al. studied the inhibitory effect of a library of ELQs on the cytochrome *bc1* complexes of *P. falciparum* and *T. gondii* parasites, having concluded that, beside ELQ-271 and ELQ-316, the very promising candidate ELQ-400 (compound (e), Figure 1) was able to inhibit both the Qi and Qo sites, possibly due to the aforementioned structural flexibility, as well as the favorable substitution pattern [40,56]. In toxoplasmosis, this compound is thought to act on both acute and chronic stages, as demonstrated by the lack of parasites in the mice tissues, including the brain, suggesting an ability of the compound to cross the blood–brain barrier as well as a strong antiparasitic effect [40]. Additionally, the compound showed better physicochemical characteristics than previously evaluated ELQs, highlighting this compound’s and the quinoline class’s striking features for further scrutinization in the context of malaria and toxoplasmosis chemotherapies and, potentially, for treating infections due to other apicomplexan parasites. 

The attractive features of the quinoline scaffold in the context of toxoplasmosis prompted Elgawad and co-workers to evaluate the effect of the quinoline-based hybrid 4-(2-chloroquinolin-3-yl)-6-(2,5-dimethoxyphenyl)-2-oxo-1,2-dihydropyridine-3-carbonitrile, also known as PPQ-8 (Figure 2), against acute and chronic toxoplasmosis mouse models [39]. The compound stood out, as it has evidenced very promising results in decreasing the overall parasitic burden and an ability to cross the blood–brain barrier, promoting the degeneration and reduction of *T. gondii* cysts containing bradyzoites, one of the major problems in toxoplasmosis, characteristic of the disease’s chronic phase. The treatment of infected mice in the acute phase with PPQ-8 led to disarrangement in the morphology of tachyzoites and consequently a significant decrease in the parasite’s distribution throughout the tissues, namely in the liver and spleen, where a considerable decrease in inflammation and histopathological changes was observed [39].

Although the authors were not able to determine the exact therapeutic target, the information available suggests a possible inhibition of the apicoplast by the quinoline core, leading to the parasite’s death, as well as a potential interference with the replicative cycle through disruption of DNA topoisomerases [39]. The latter may possibly be justified by the resemblance of PPQ-8 with antibacterial fluoroquinolones, known as topoisomerase inhibitors, which in fact were shown to exhibit high efficacies and survival rates in acute-phase infected mouse models of toxoplasmosis and also in some *P. falciparum* models [19,57].

#### 2.2.3. Artemisinin and Related Endoperoxides

**Figure 3:**
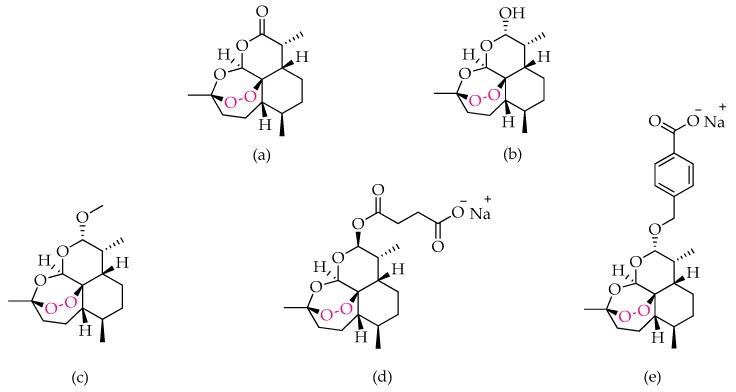
Structural representation of endoperoxides used as antimalarials: artemisinin (**a**), dihydroartemisinin (**b**), artemether (**c**), artesunate (**d**), and artelinate (**e**), highlighting the peroxide bond.

As for the quinoline class, the first use of endoperoxides against parasitic illnesses emerged from the discovery of the antimalarial properties in *Artemisia annua*, a herb used in traditional Chinese medicine [58,59]. Artemisinin (structure (a), Figure 3), a sesquiterpene lactone that contains a peroxide bridge essential for its activity, is one of a very limited number of naturally occurring endoperoxides and was shown to inhibit chloroquine-resistant strains of *Plasmodium falciparum* parasites at low nanomolar concentrations [60], with efficacies comparable to those exhibited by chloroquine and mefloquine when tested against the corresponding sensitive strains. Artemisinin was initially used to treat malaria but limitations, such as low solubility in both water and oil, a short half-life, and its fast metabolization to dihydroartemisinin, DHA, (structure (b), Figure 3), which is neurotoxic, demanded for more soluble and less toxic semi-synthetic derivatives, commonly known as artemisinins [61,62,63]. Additionally, low yields, high production costs, and inefficacy against all strains of *Plasmodium* prompted the development of synthetic endoperoxides [62,63,64,65]. Nevertheless, artemisinin and some of its semi-synthetic derivatives, namely DHA, artemether, artesunate, and artelinate (represented as structures b, c, d, and e in Figure 3, respectively), when used in combination with other longer-lived antimalarials, provide the artemisinin-based combination therapy (ACT), which is used as frontline treatment for malaria [6].

Recent data shows that artemisinin and its derivatives also exhibit activity against *T. gondii* [27,66,67,68,69,70,71], yet different potencies reinforce the possibility of different targets being affected in each case and/or differences in drug accumulation among different parasite species. While in *Plasmodium* it has been proposed that endoperoxides may have an important role in heme detoxification and the production of reactive species mediated by the host’s heme iron, contrasting studies in *T. gondii* appear to indicate that the main mechanism of action of this class of compounds in *T. gondii* is associated with their interference with calcium homeostasis, affecting all parasitic processes that are dependent on this mineral [65,72,73,74]. 

**Figure 4:**
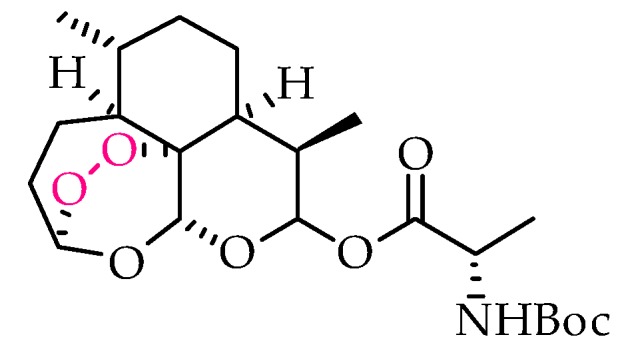
Structural representation of the antitoxoplasmic artemisinin derivative A2, studied by Deng’s group and selected as a lead for further development.

Regarding the activity of endoperoxides against toxoplasmosis, both acute and chronic phases were shown to be affected, although potencies in some cases fell short and were rather variable among the tested derivatives [66,67,68,69,70,71]. Recently, Deng and colleagues performed in vitro and in vivo studies to evaluate the activity of a library of artemisinin derivatives and their selectivity towards the parasite [67], with the in vitro data demonstrating that all derivatives tested exhibited better selectivity than the controls DHA and spiramycin. From the library investigated, compound A2 (Figure 4) emerged as the most promising lead, showing the highest selectivity in vitro and also a very promising inhibition of the acute-phase tachyzoites in vivo, since its action significantly decreased invasion of the peritoneal cavity in mice and led to a reduction in hepatic and splenic inflammatory processes [67]. These results show that antimalarial endoperoxides and their derivatives undoubtedly deserve further investigation as possible antitoxoplasmic drug candidates.

#### 2.2.4. Pyrazolo[1,5-*a*]pyrimidines

**Figure 5:**
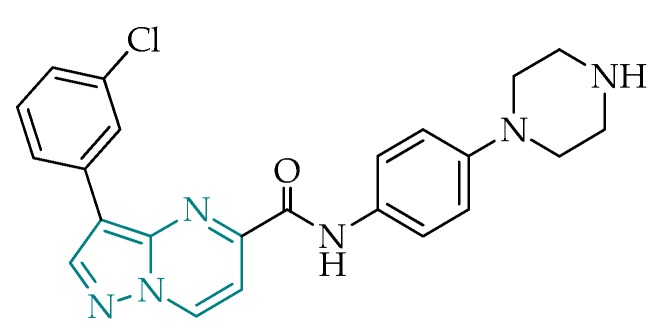
Structural representation of pyrazolo[1,5-*a*]pyrimidine MMV022478, identified by Spalenka’s group [31], highlighting the pyrazolo[1,5-*a*]pyrimidine frame.

Pyrazolo[1,5-*a*]pyrimidines are a class of structurally challenging compounds with interesting properties [75], including promising antibacterial activity, as seen, for example, in preliminary studies against *Mycobacterium tuberculosis* [76,77], as well as antiparasitic activity against *Plasmodium spp.* [78,79] and other protozoans, such as *Trypanosoma* spp. and *Leishmania* spp. [80,81]. In addition, studies conducted by Spalenka et al. led to the identification of a pyrazolo[1,5-*a*]pyrimidine, shown to attain antitoxoplasmic activity at low micromolar inhibitory concentrations, with high selectivity towards the parasite [31]. The work group evaluated a series of antimicrobial derivatives from the pathogen box, and pyrazolopyrimidine MMV022478 (Figure 5) emerged as one of the most encouraging candidates for *T. gondii* inhibition. This compound was selected based on structural similarities with the currently available drug pyrimethamine, indicating that the suspected drug target is the dihydrofolate reductase enzyme (DHFR). These findings revealed positive features of the pyrazolo[1,5-*a*]pyrimidine scaffold and stimulated research for this class as potential selective DHFR inhibitors in *T. gondii*.

#### 2.2.5. Immunomodulatory Agents

##### Antimicrobial Peptides

Increasing incidence rates of drug resistance in many therapeutic fields, namely bacterial and parasitic diseases, stimulated the search for different sources of therapeutic agents against these diseases to circumvent current difficulties. Antimicrobial peptides (AMPs) are a class of compounds that emerged as promising antimicrobial alternatives to present chemotherapies [82,83,84]. These molecules are very versatile, having numerous activities that include antiviral, antifungal, anti-mitogenic, anti-cancer, and anti-inflammatory [85]. Morphologically, AMPs are the first defense elements of the innate immune system produced by a variety of organisms, including humans, and they are responsible for the elimination of intruder entities through the disruption of cytoplasmic membranes, leading to cell damage and death, as well as immunomodulation through activation or suppression of key immunomodulatory biomolecules [82,83,84,86,87]. Recent studies have highlighted the activity of such peptides against parasitic diseases, with malaria being one of the leading parasitic illnesses researched in this field [88,89,90,91,92,93,94,95,96,97,98,99]. New data incorporating AMPs have also surfaced as a promising source of antitoxoplasmic candidates [97,100,101,102,103,104,105,106,107].

The majority of the studied AMPs act on tachyzoites, i.e., on the proliferative stage of *T. gondii,* inhibiting this process through direct killing of the parasite in the bloodstream and by reducing primary cell infection. Although these are aimed solely at an acute phase of the disease, some results show better activities than currently available drugs pyrimethamine, sulfadiazine, or spiramycin [105,106,107]. Lee et al. studied the activity of antimicrobial peptide interferon (IFN)-γ–inducible immunity-related GTPase (Immunity-related GTPase, IRG protein) on the intracellular reproductive stage of *T. gondii* tachyzoites by acting on the parasitophorous vacuole [108], having found that IRG proteins are relevant effector agents in the process of vacuole disruption and breakdown, reinforcing the multifarious character of antimicrobial peptides and their potential use in the therapy of toxoplasmosis, targeting multiple stages of the cycle, and bypassing the parasite’s ability to acquire sophisticated mechanisms of drug resistance.

**Scheme 1:**
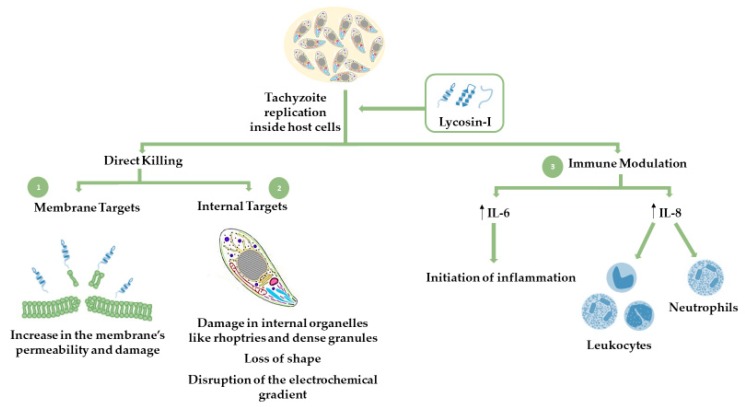
Schematic representation of the mechanism proposed for the action of antimicrobial peptide (AMP) lycosin-I on *T. gondii*-infected host cells.

Lycosin-I is a cationic peptide derived from the venom of the spider *Lycosa singorensis*. This peptide acts as a potent inhibitor against inflammation, malignant tumors, and microorganisms, essentially affecting cells carrying charged cytoplasmic membranes. Recent studies have shown that lycosin-I is also effective against infections with *T. gondii* and acts through disruption of the cell plasma membrane [58,59]. The interaction between lycosin-I and the negatively charged intruder cell mediates the disruption of the membrane, exposing the compound to the intracellular environment, where it can concomitantly affect internal structures and lead to organelle damage, loss of shape, vacuolization, and breakdown [58,59]. Additionally, this molecule was shown to inhibit intracellular proliferation of tachyzoites, with inhibition rates higher than those observed in treatments with sulfadiazine [109]. Apart from the direct mechanism of inhibition, lycosin-I was also shown to activate an immune response through an increase in the production of interleukins IL-6 and IL-8 cytokines [109]. Scheme 1 depicts the inhibition pathways mediated by lycosin-I.

##### Fungal Secondary Metabolites

Secondary metabolites are organic compounds produced by a variety of organisms, including fungi, bacteria, and plants. Fungi represent a valuable source of secondary metabolites with impact in anti-infectious therapies and can be classified into four distinct groups: Cyclic depsipeptides, peptaibiotics, non-depsipeptide cyclic lipopetides, and non-peptaibiotic linear lipopeptides. Similar to AMPs, these compounds act as defense mechanisms against external stimuli [110,111]. Cyclic depsipetides are part of a group enriched with many therapeutic properties, including antimicrobial, antioxidant, and immunomodulatory and are also inhibitory of several biomolecules and biomolecular processes, such as the activity of α-glucosidase, cholesterol acyltransferase, xanthine oxidase, and platelet aggregation [111]. Promising antiprotozoal activities were also observed for compounds belonging to this class, both against malaria and toxoplasmosis [110,112]. A library of compounds isolated from the beauvericin-like family of the cyclic depsipeptides exhibited activity against *Plasmodium falciparum* within the IC50% range 0.24–3.4 μg/mL [110].

**Scheme 2:**
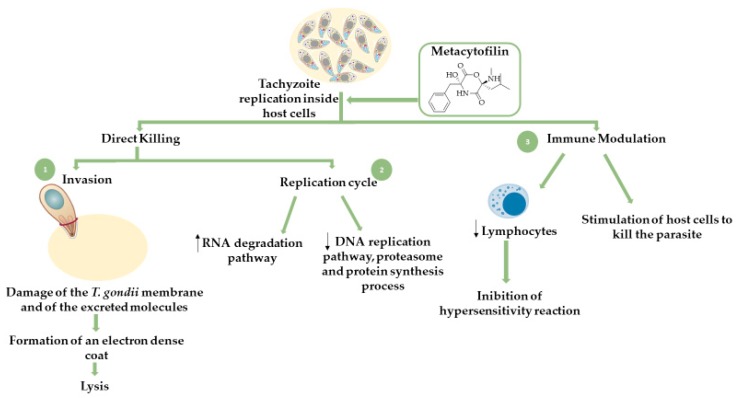
Schematic representation of the mechanism proposed for the action of metacytofilin on *T. gondii*-infected host cells.

Metacytofilin (structure illustrated in Scheme 2) is a natural secondary metabolite produced by *Metarhizium fungus* and belongs to the depsipetides’ group, more specifically cyclic didepsipeptides, which are characterized by possessing an ester group and an amide group in the same six-membered ring [111,113,114]. This compound showed favorable results regarding its activity against *T. gondii* parasites. Analogously to AMPs, metacytofilin acts through a dual mechanism, targeting both the parasite directly through inhibition of its replicative cycle and the host immune system through suppression of the inflammatory processes and stimulation of immune cells to combat the infection [112]. A detailed mechanism of action of this compound can be seen in Scheme 2.

It is also important to note that three main strains of *T. gondii* have been identified, type I, II, and III, which differ in virulence and the epidemiological pattern of occurrence [12]. The most common strains affecting humans, namely immunosuppressed patients and those with congenital toxoplasmosis, are types I and II [12]. Metacytofilin was shown to inhibit the intracellular growth of both type I and type II strains [112]. Additional advantageous properties of metacytofilin include a favorable pharmacokinetic profile, i.e., good gastrointestinal absorption, allowing for oral administration, which in turn translates into low production and storage costs, as well as privileged properties for distribution and administration.

## 3. Conclusions

Toxoplasmosis is a neglected infectious disease, mostly associated with disadvantaged populations. The chemotherapeutic tools used to combat toxoplasmosis, in addition to not being exclusively used for this infection, show reduced efficacy, due to the fast development of resistance by the parasite, and, in most cases, toxicity to the host and high cost. In recent years, the scientific community has intensified efforts in the search for new drugs for the treatment of toxoplasmosis that act in both the chronic and acute phases of the disease, display safer profiles, and are able to reach organs, such as the brain and the eyes. The knowledge gathered through intense research on *Plasmodium* spp. and malaria allowed for the discovery of many therapeutic solutions that are useful not only in the treatment of malaria but also as tools to fight other infectious diseases caused by protozoan parasites that share parasite similarities and metabolic pathways, as is the case of toxoplasmosis. New studies have spurred the identification of new pharmacological candidates, active on both *Plasmodium* and *T. gondii* parasites, highlighting classes like quinolones, endoperoxides, and pyrazolo[1,5-*a*]pyrimidines, among others. Further research should therefore be undertaken with upmost urgency, in the hope that, in the future, these results will promote an increase in the therapeutic arsenal for toxoplasmosis and malaria, eliminating the gaps in the current chemotherapy.

## Figures and Tables

**Table 1:** Chemotherapeutic approaches commonly used for toxoplasmosis.

Type of Disease	Front-line Treatment	Alternatives
Congenital toxoplasmosis	Pyrimethamine + Sulfadiazine + Folinic acid	Spiramycin for prophilaxis
Cerebral toxoplasmosis		Co-trimoxazole or Atovaquone
Retinocoroiditis		Prednisone as coadjuvant; Clindamycin; Co-trimoxazole; Atovaquone
